# Just-in-Time Adaptive Intervention to Promote Walking Behavior and Reduce Stationary Time in Physically Inactive Adults: Protocol for the Walking With JITAIs Study

**DOI:** 10.2196/79022

**Published:** 2026-01-07

**Authors:** Cora J Firkin, Ajith Vemuri, Tanvir Rahman, Barry Bodt, Elizabeth Orsega-Smith, Keith Decker, Gregory M Dominick

**Affiliations:** 1 Department of Health Behavior and Nutrition Sciences College of Health Sciences University of Delaware Newark, DE United States; 2 Department of Computer and Information Sciences College of Engineering University of Delaware Newark, DE United States; 3 Biostatistics Core Facility College of Health Sciences University of Delaware Newark, DE United States

**Keywords:** Apple Watch, behavior change, feasibility, iPhone, just-in-time adaptive intervention, physical activity, wearable, mobile phone

## Abstract

**Background:**

A Just-in-Time Adaptive Intervention (JITAI) recognizes the dynamic nature of individuals’ states and contexts, predicts support needs, and sends tailored support at more opportune, actionable times.

**Objective:**

This paper outlines the application architecture and protocol for the pilot “Walking With Just-in-Time Adaptive Interventions” (WWJ) study, which uses a JITAI approach to improve walking behavior—duration, speed, and distance—and reduce stationary time, defined as idle sitting or standing.

**Methods:**

This study targets 20 adults who are physically inactive and leverages the Apple Watch to deliver fully automated tailored intervention notifications to “walk faster,” “walk longer,” or “stand up and move around” based on real-time data and contextual factors, including time-of-day activity patterns, geographic locations (eg, home, work, park, and gymnasium), weather conditions (eg, precipitation, wind speed, and humidity), and receptiveness. The protocol involves a preintervention assessment of demographics, behavior change constructs, anthropometrics, and resting vital signs; a 2-week observation period to establish walking behavior and stationary time baselines; a 2-week just-in-time learning period to evaluate receptiveness to untailored prompts at all applicable times; the 2-week JITAI intervention phase; and a postintervention assessment. Feasibility will be evaluated through protocol fidelity, participant adherence, Apple Watch wear-time compliance, user burden, acceptability ratings, and perceptions of benefits and preferences.

**Results:**

The WWJ architecture development began in spring 2021 and concluded in fall 2022. Participant recruitment and enrollment began in fall 2022. A total of 18 participants were recruited. Upon completion of the analyses, the results of this study are expected to be submitted for publication.

**Conclusions:**

Distinctively, the WWJ just-in-time learning period aims to train the learner based on user receptiveness within contexts by sending interventions whenever a participant meets the predetermined thresholds regardless of the likelihood that the user will be receptive to the notification to prune out nonopportune or “nonactionable” times. This approach may allow for greater customization during the JITAI period.

**International Registered Report Identifier (IRRID):**

DERR1-10.2196/79022

## Introduction

### Background

Regular physical activity (PA) participation attenuates the accumulation and progression of cardiometabolic risk factors such as hypertension, dyslipidemia, overweight and obesity, and insulin resistance that contribute to cardiometabolic disease morbidity and mortality [1,2]. PA, broadly defined as “any bodily movement produced by skeletal muscles that results in energy expenditure,” [3] is characterized by 4 dimensions: duration, frequency, intensity or rate of energy expenditure (EE), and PA type [3]. Historically, the 2007 American Heart Association Guidelines for Americans suggested adults 18-65 years of age should participate in moderate-intensity physical activity (MPA) for a minimum of 30 minutes per day for 5 days per week, including only intermittent bouts lasting 10 or more minutes [4]. The 2020 World Health Organization (WHO) recommendations build upon these guidelines, suggesting that the participation in 150-300 minutes of weekly MPA, 75-150 minutes of weekly aerobic vigorous-intensity physical activity, or an equivalent weekly combination of aerobic moderate-to-vigorous-intensity physical activity (MVPA) can be accumulated in bouts which can be of any length [5]. This approach allows for more flexibility in the accumulation of MVPA throughout each day, in which each minute counts toward weekly participation [5]. Nonetheless, a substantial proportion of adults—approximately 30% worldwide and 42% in high-income Western countries—fail to achieve these MVPA recommendations [6].

Moreover, a 2018 review found that adults spend approximately 8 hours participating in sedentary behavior (SB) daily [7]. SB is characterized by EE less than or equal to 1.5 metabolic equivalents of task (MET), with the Sedentary Behavior Research Network further detailing in 2017 that SB occurs while in a seated, reclined, or lying posture [8]. Emerging evidence indicates that excessive SB participation is associated with an increased prevalence of cardiometabolic diseases and risk of all-cause mortality [9] while reducing SB participation and subsequently increasing PA participation—even to a light-intensity—can significantly improve BMI and diastolic and systolic blood pressure (BP) [10-12]. Additionally, while intermittent standing bouts may be a feasible approach to reduce prolonged sitting, reclining, or lying through a postural change, passive or “idle” standing produces limited bodily movement, typically remaining below the 1.5 MET threshold to classify as light-intensity physical activity (LPA) [13].

Thus, reducing stationary behavior (ie, SB and idle standing) is a public health challenge that should be addressed through movement behaviors that reach MET thresholds at or above an LPA level. While specific LPA guidelines have yet to be established, the WHO advises adults to minimize SB time and substitute that time with any type and intensity of PA, including LPA [5]. Given that walking is generally feasible and accessible for adults who ambulate [5,14], it can serve as an ideal mode for not only replacing stationary behavior with LPA participation but also meeting WHO MVPA recommendations [5,14,15]. Walking paces around 0.82-0.89 m/s can exceed the 1.5 MET threshold for LPA [16,17]. To achieve the MPA threshold, walking must be performed at faster paces, typically around 1.3-1.8 m/s [16]. Since the 2020 WHO recommendations recognize that every minute of MVPA—regardless of bout duration—contributes toward an individual’s weekly targets [5], promoting walking behavior at faster paces may be an appropriate approach to increasing weekly MVPA volume while also breaking up stationary behavior.

### Mobile and Wearable Technology

Mobile and wearable technology advancements have unlocked innovative opportunities for collecting real-time walking and stationary behavior data and delivering individualized, theoretically driven, evidence-based interventions [18]. This technology can impact human behavior within “real-world” contexts by delivering actionable notifications based on real-time data collected from a user’s smartphone or wearable device [19,20]. A market-leading consumer-grade wearable—the Apple Watch (AW)—is equipped with a range of features for real-time data collection and notification interaction. Features include triaxial accelerometer, gyroscope, heart rate (HR), and GPS sensors that use Bluetooth to transmit and receive data and interact with other devices or cloud-based services. Notably, systematic reviews have supported the promising validity, reliability, sensitivity, and feasibility of leveraging the AW for assessing PA and SB [21,22]. Prior work has established that the AW can classify movement types (eg, sitting, lying, and walking) with moderate accuracy [20]. With the ability to dynamically detect behavioral and contextual variables in real-time and interact with users through immediate notifications, the AW is a feasible platform for delivering behavioral interventions, such as just-in-time and Just-in-Time Adaptive Interventions (JITAI), to increase walking behavior and decrease stationary time [19,20].

### Just-in-Time Intervention

According to the literature, a just-in-time behavioral intervention approach provides real-time support that is typically delivered through mobile or wearable applications or SMS text messaging or “texting” and is tailored to the user’s current behavioral, psychological, and physiological state using data via smartphone or wearable sensors [23,24]. This support typically encourages users to further engage in positive, goal-directed health behaviors or discourages them from further engaging in their current behaviors, such as prolonged SB [23,24]. Additionally, some just-in-time interventions have provided notifications only once daily, typically triggered at a preset time or event (eg, 10 AM or postgoal achievement), focusing mainly on summarizing past performance to motivate future behavior, rather than offering continuous or actionable notification [25,26]. The preprogrammed health and fitness notifications of the AW exemplify this approach by providing stand “reminders” or feedback—cuing users to move every 50 minutes if prolonged stationary behavior without at least a minute movement break was detected—and behavioral reinforcement through celebratory feedback messages when daily goals are met by closing the “move,” “exercise,” and “stand” rings which indicate aerobic EE, MVPA participation, and frequency of stationary breaks. Nevertheless, these notifications follow a rigid, predefined schedule or pattern rather than dynamically responding to the user and adapting to the various contextual influences that may impact their current behavior (eg, weather or geographic location) [24]. Thus, just-in-time interventions may have a limited impact on behavior change because they do not learn from and adapt to users’ states and their various contextual influences, remaining more prescheduled or reactive than predictive and personalized over time.

### About JITAI

To determine the most opportune moments for behavior change, JITAI approaches monitor changes in users’ behavioral, psychological, and physiological states, and environmental contexts over time to deliver support given the user’s state of need and receptivity to real-time actionable notifications [23,24]. This approach recognizes the dynamic nature of individuals’ states and contexts, requiring the system to predict (1) whether the person requires support, (2) the type or amount of support needed, and (3) whether or not the support will be acted upon [23]. A 2025 scoping review of JITAIs across several health behaviors (eg, PA, SB, fluid intake, and substance use) and settings revealed that while the included JITAIs were deemed feasible, a majority (N=62, 55%) of approaches solely relied upon self-reporting data, with few studies (21%) collecting passively monitored data via sensors in real time [27]. A 2019 systematic review of JITAIs specifically designed to promote PA and reduce SB participation revealed mixed evidence on their effectiveness [24], emphasizing notable feasibility concerns, such as low adherence and acceptability, and lack of a strong theoretical basis to guide the JITAI approaches or explain behavior changes. Reviews have highlighted the favorable use of the social cognitive theory and transtheoretical model (TTM) in the development and evaluation of mobile and wearable technology-based interventions for promoting PA and reducing SB [18,25,28,29]. To refine and optimize the effectiveness of the JITAI implementation for health behavior change, a thorough evaluation of the feasibility of the JITAI approach and the relevance of social cognitive theory and TTM behavior change constructs is warranted [18,24].

### Objective, Aims, and Research Questions

This protocol paper presents the development of the “Walking With Just-in-Time Adaptive Interventions” (WWJ) iPhone and AW app, server, and methodology for a 6-week pilot study among adults who are not meeting the 2020 WHO PA recommendations. The primary aims of the WWJ’s study are to examine the feasibility and initial impact of a 2-week JITAI approach for increasing walking behavior (ie, walking speed, distance, and duration) and decreasing stationary behavior (ie, duration of SB and idle standing) compared to a 2-week baseline observation period and a 2-week just-in-time learning period. Thus, our corresponding research questions include: (1) Is the WWJ feasible, regarding recruitment, retention, intervention fidelity, acceptability, adherence, and engagement? (2) To what extent does the just-in-time approach impact walking and stationary behavior compared to baseline? (3) How do changes in walking and stationary behaviors across a 2-week just-in-time and a 2-week JITAI approach compare to baseline?

## Methods

### Overview of Study Design

**Figure 1:**
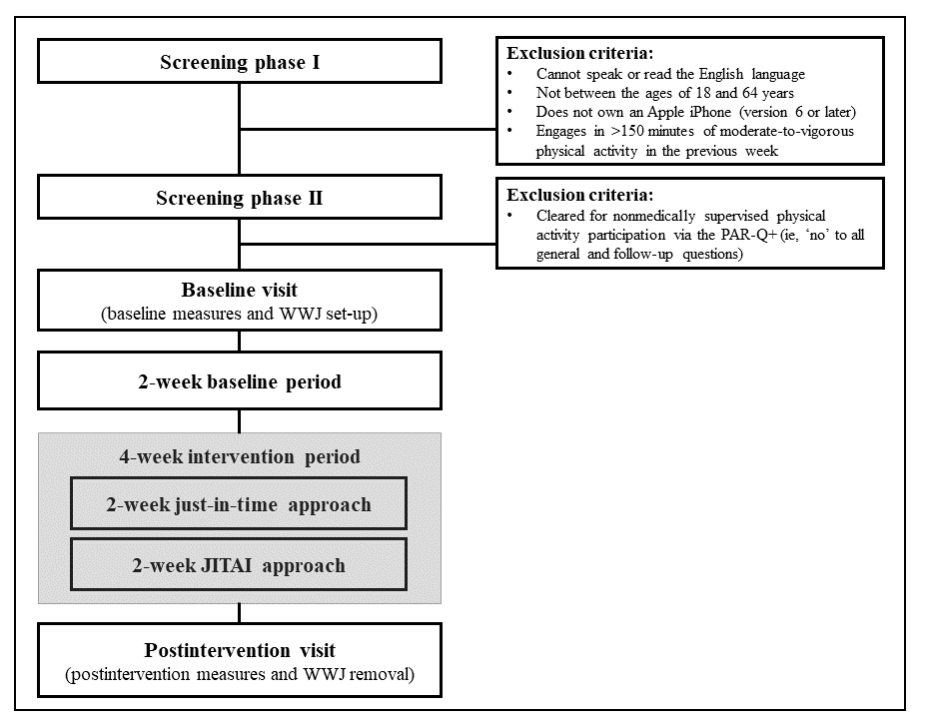
CONSORT diagram: the WWJ study approach. CONSORT: Consolidated Standards of Reporting Trials; JITAI: Just-in-Time Adaptive Intervention; PAR-Q+: Physical Activity Readiness Questionnaire for Everyone; WWJ: Walking With Just-in-Time Adaptive Interventions.

This pilot feasibility study will use a 1-way within-subjects repeated-measures design to examine changes in walking and stationary behaviors across the 3 time periods: baseline, just-in-time, and JITAI. This study will consist of a preintervention measurement session, followed by a 2-week observation period that will serve as a baseline for walking and stationary behaviors, a 2-week just-in-time learning period, a 2-week JITAI period, and concluding with a postintervention measurement session (Figure 1). The receptiveness-in-context data from the just-in-time learning period will be used to train and individually adapt the JITAI server by learning the ideal contexts in which the participants are most receptive to each of the 3 intervention notifications, as detailed in the Architecture of the WWJ App and Server section.

### Participant Eligibility and Recruitment

A total of 20 participants will be recruited through community and clinical settings within a small city in New Castle County, Delaware, United States, using flyers, referrals, and social media (eg, Facebook and Instagram). Prospective participants will be directed to an online screening portal for the first screening phase (Figure 1) to determine study eligibility. Inclusion criteria will be adults aged 18-65 years, who own an Apple iPhone version 6s or newer, can speak and read in the English language, and self-report not achieving the minimum MVPA threshold of 150 weekly minutes in the previous week [5]. Exclusion criteria in the second screening phase will include answering “yes” to any general health and follow-up questions from the Physical Activity Readiness Questionnaire for Everyone, indicating that they are not cleared for PA participation without medical consultation and supervision [30]. If the screener indicates that a prospective subject is eligible, the informed consent form will be delivered to prospective participants via REDCap (Research Electronic Data Capture, Vanderbilt University).

### Architecture of the WWJ App and Server

**Figure 2:**
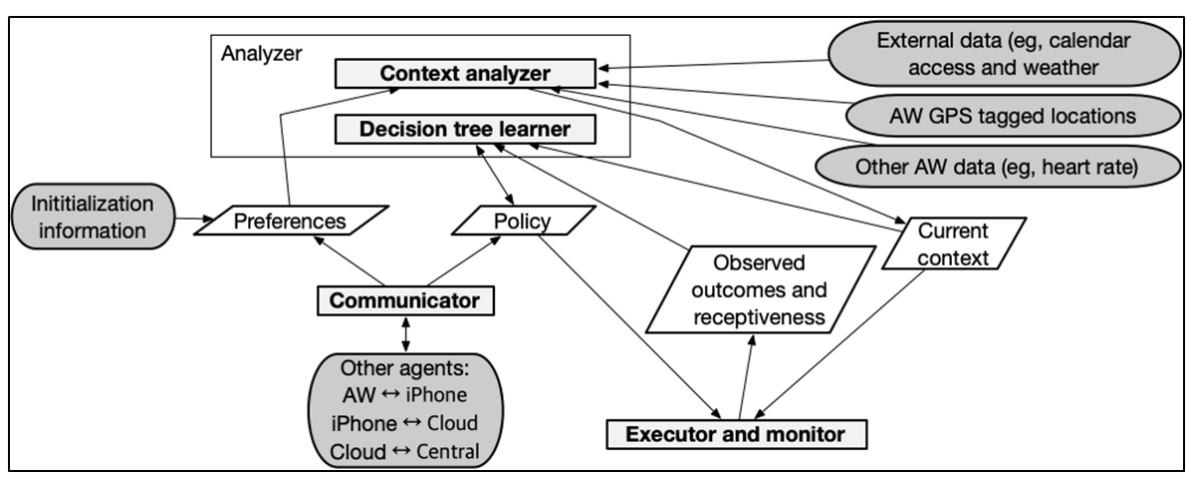
WWJ architecture per agent. AW: Apple Watch; WWJ: Walking With Just-in-Time Adaptive Intervention.

The research team has previously detailed a comparable server and application development [19]. The WWJ fully automated multiagent system operated on a per-agent architecture comprising 4 modules—context analyzer, learner, executor, and communicator (Figure 2). In the WWJ agent-level design, learning the support need policy is framed as a fully supervised classification problem rather than as a Markov Decision Problem [31] to account for the time constraints of a 2-week baseline and 2-week just-in-time learning period and the AW’s battery-life constraints, which are particularly so when leveraging the HR sensor and GPS networks. Architecture development began in spring 2021 and concluded in fall 2022. Aligning with the CONSORT EHEALTH checklist (V.1.6.1), all updates to the WWJ application and server after this finalized development will be identified by date, version number, and description to allow for the replicability of the intervention [32].

**Figure 3:**
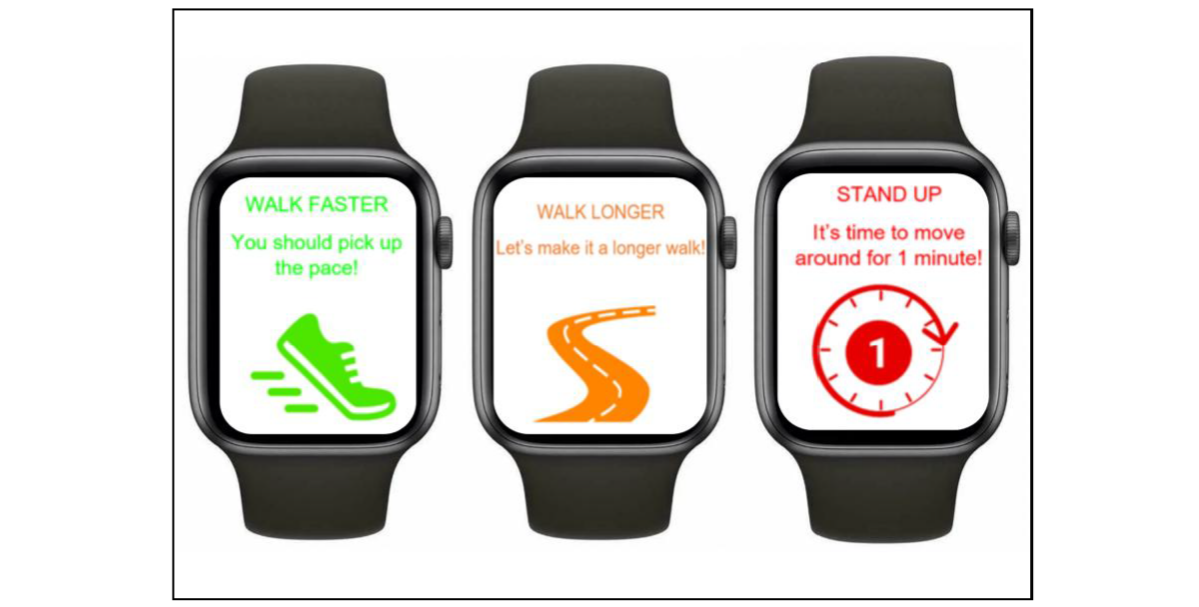
The WWJ intervention notifications: walk faster, walk longer, and stand up. WWJ: Walking With Just-in-Time Adaptive Intervention.

The context analyzer synthesizes external (eg, weather and calendar) and internal (eg, GPS coordinates mapped to tagged locations) contexts into a current context feature vector. At decision points, these contextual features (detailed under “contextual variables”) are used in binary classification models to determine whether an intervention notification should be sent based on the predicted likelihood of successful behavior change within these contexts as processed and interpreted by the learner module. Thus, 3 decision trees (policies) will be trained for each user corresponding to the intervention notification types (Figure 3)—walk faster (ie, “You should pick up the pace”), walk longer (ie, “Let’s make it a longer walk”), and stand up and move around (ie, “It’s time to move around for one minute”).

The learner module (Figure 2) also establishes baseline walking and stationary behaviors during the initial 2-week observation period by passively monitoring behaviors and calculating their applicable averages (eg, average walking speed) per bout. During the just-in-time learning period, intervention notifications are triggered at each instance that participants meet a predetermined threshold for walking and stationary behaviors—unless they have marked themselves as “busy” on their calendar to not burden the user during unsuitable times such as work meetings. The threshold for the walking notifications is set at 250 m from the initial start point of a walking detection. This distance was chosen after trial-and-error field testing among 4 authors (CJF, AV, KD, and GMD), which indicated that walking less than 250 m from a given starting location may not align with viable opportunities to walk faster or longer. The WWJ application will send a “walk longer” notification if the system recognizes that the user is in or near a previously tagged location. In all other cases, participants will receive a “walk faster” notification. When stationary behavior is detected for 30 continuous minutes, the application will send the “stand up and move around” notification. The system will determine if these interventions are successful or not (ie, receptiveness) either by comparing the current behaviors in the just-in-time learning period to those in the observation period within the same contexts for walking behavior or by the AW detecting that the user got up and walked around for a minute for stationary behavior. As shown in Figure 2, data collected by the executor during the just-in-time learning period serves as labeled training data to compute and optimize personalized prompting policies for the JITAI period. At the end of the just-in-time learning period, the learner module uses this labeled data to generate a decision-tree–based policy, predicting when and when not to send the intervention notifications to the user based on their current context, past receptiveness, and predicted support needs. As predicting behavior is uncertain, the learning model will deliberately use intervention notifications even if the user may not be receptive, with a 10% chance of such occurrences, to allow behavior change in previously nonoptimal contexts and for the system to learn continually.

**Figure 4:**
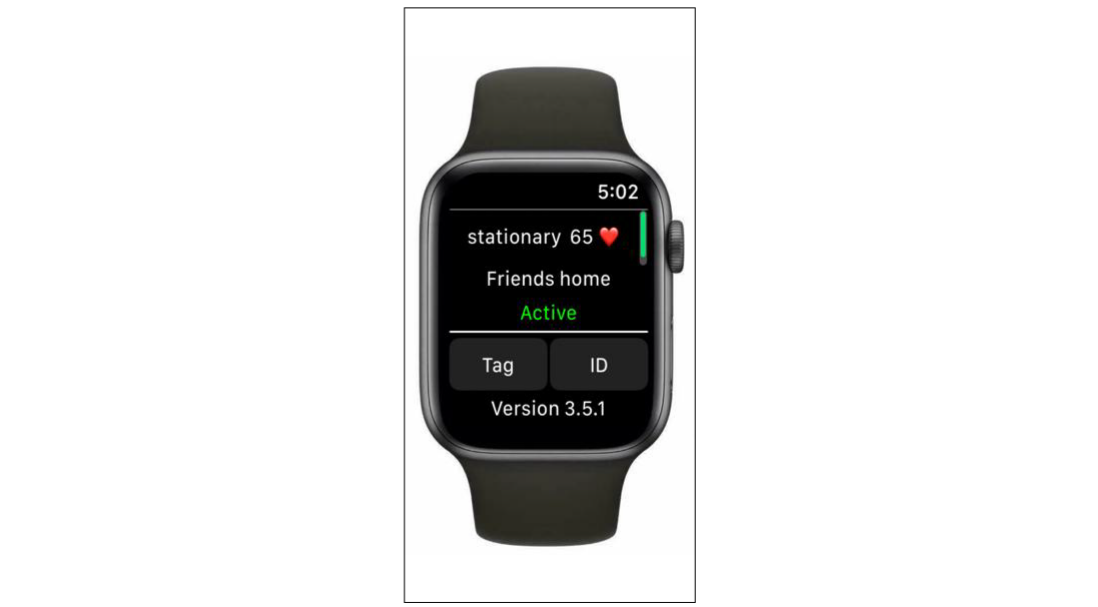
The WWJ application’s main user interface. WWJ: Walking With Just-in-Time Adaptive Intervention.

The communicator module will ensure that data will flow seamlessly through connections between the AW and a paired iPhone, the iPhone and Cloud, and the Cloud to our central server for secure data storage, processing, and offline analysis. This transmission process will occur every 2 hours. This timeframe was chosen after the aforementioned trial-and-error field tests to maximize AW battery life. When the WWJ application is running on the AW, the main user interface displays the current behavior (stationary or walking), HR, and tagged location of the participant (Figure 4).

### Measures and Materials

#### Apple iPhone and Watch Device Compatibility and Software

To ensure compatibility with the WWJ application, participants will use an iPhone 6s or newer running iOS 13 or later and an AW Series 3 or newer running watchOS 8.0 or later. If the participant does not own an AW Series 3 or is newer, they will sync a borrowed AW Series 3 running watchOS 8.0 or later with their iPhone. All applicable updates will be conducted for optimal performance. We will monitor iOS and watchOS updates to identify any potential impacts on just-in-time and JITAI notifications and address any issues that arise.

#### Demographics

Demographics will be collected using an adapted survey based on the demographics section of the 2021 Centers for Disease Control and Prevention–approved Behavioral Risk Factor Surveillance System Survey Questionnaire [33] on the following items: age; sex; race and ethnicity; level of education; employment, marital, and student statuses; annual household income; current housing arrangement (ie, renting, owning, or in a dormitory); and current ZIP code.

#### Behavior Change Constructs

The stages and processes of PA change from TTM will be assessed using scales developed by Marcus et al [34]. Additionally, the 5-item Self-Efficacy for Physical Activity scale will assess an individual’s confidence in engaging in PAs despite barriers, such as tiredness, bad mood, time constraints, vacation time, and unfavorable weather conditions, that is, rain or snow [34]. PA self-regulation will be assessed using the 12-item version of the Physical Activity Self-Regulation scale, which has excellent internal consistency (Cronbach α=0.91) and good construct validity [35].

#### Anthropometrics and Resting Vital Signs

Body composition will be estimated using BMI (ie, body weight [in kg] divided by height [in m^2^]). Participants will remove their shoes and any excessive clothing for all measurements. Weight will be measured using a calibrated, portable Seca scale to the nearest 0.1 kg. Height will be measured using a calibrated, portable stadiometer to the nearest 0.5 cm. Using a standard stopwatch, radial resting HR will be measured for a full 60 seconds to the nearest beat after the participant is instructed to sit in a relaxed position in a chair with their back supported, feet on the floor, and arms supported at heart-level for 5 minutes in a private room [36]. Resting systolic and diastolic BP will be measured to the nearest mm Hg by a trained researcher using a standard portable BP machine (OMRON Intelli-Sense Series 7 Advanced Accuracy, Model BP760) and cuff size that allows for the bladder of the cuff to encircle at least 80% of the upper arm. The researcher will wrap the cuff firmly around the upper left arm of the participant at heart level and align the cuff correctly with the brachial artery. A second reading for resting HR and BP will be taken after an additional 1-2 minutes. If the BP readings are 5 or more mm Hg apart, a third reading will be obtained after a final 1-2 minutes of rest [36].

#### Contextual Variables

##### Busy Times of Day

During the baseline visit, participants will sync all their calendar systems (eg, Google, Microsoft Exchange, and Outlook.com) to their Apple Calendar on their paired iPhone. They will be instructed to use the Apple Calendar to schedule times they cannot be notified during by marking those events as “busy.” Participants will be advised not to mark large blocks of time (eg, a full 8-hour workday) as “busy” and to ensure all scheduled physically active times are marked as “free.”

##### Geographic Location

Participants will be instructed to provide a text label of their physical locations via the “tag” button on the WWJ user interface the first time they visit a new location while they are in that precise location (eg, tagging work while seated at one’s work desk). After pushing the “tag” button, participants will have the option of selecting home, work, school, gymnasium, park, and other; if the user selects “other,” they can type in any new text label (eg, “friend’s house” as illustrated in Figure 4). Instructions will be given not to tag: (1) a location in a parking lot, (2) a nonprecise or overly generic location (eg, university campus versus a specific university building), or (3) behavior they are participating in (eg, walking around the neighborhood). Once tagged, the AW system will register each instance when the participant is in this location with a 50-m accuracy whenever data are recorded.

##### Weather

The ClimaCell application programming interface (API) of watchOS (currently Tomorrow.io API; Tomorrow Companies Inc) will collect participant data on precipitation to the nearest inch, temperature to the nearest Celsius degree, humidity to the nearest percent, and wind speed to the nearest mile per hour at the participant’s current precise geographic location, leveraging detected latitudes and longitudes on the AW, with a 50-m accuracy during walking and stationary bouts.

##### Walking and Stationary Behaviors

Our WWJ app will collect timestamped walking and stationary behaviors once respective thresholds are met (described previously in the Architecture of the WWJ App and Server section). The Apple watchOS behavior detection APIs use its combined location sensors—the GPS, the Globalnaya Navigatsionnaya Sputnikovaya Sistema, Galileo, and Quasi-Zenith Satellite System—and its motion sensors—triaxial accelerometer and gyroscope—to detect walking and stationary behaviors via Apple’s proprietary algorithms. These walking behaviors will include duration to the nearest whole second, distance to the nearest meter, and speed calculated from the collected distance divided by duration to the nearest meter per second. In addition, when a walking bout is detected, HR will be collected every 10 seconds. The duration of stationary behavior will be recorded to the nearest whole second.

##### Feasibility

The feasibility assessment of the WWJ pilot study will be guided by the recommendations outlined by National Institutes of Health’s National Center for Complementary and Integrative Health [37]. Key feasibility metrics will include rates of prospective participants screened per month, participants enrolled per month, retention of participants per study measure, fidelity for protocol delivery, and adherence to study protocol. In cases of nonadherence to the WWJ intervention notifications, a follow-up notification will be sent to the participant to inquire why the prompt was not followed [18]. Response options will include “I did not see the notification,” “I did not want to respond,” “It was not a good time,” and an option to choose “other” for inputted responses. Additionally, compliance with wearing AW for at least 10 hours each day for at least 4 hours each week will be determined. An exit survey will include perceived user burden with items adapted from the 2016 User Burden Scale developed by Suh et al [38], acceptability, treatment-specific expectations of benefits, and treatment-specific preferences for consideration for future iterations of WWJ to further detail user feedback [18].

### Protocol

#### Preintervention Visit and WWJ Application Set-Up

The preintervention visit will take place in a private room to explain study procedures and complete baseline assessments (ie, demographics, behavior change constructs, anthropometrics, and resting vital signs). The AW will be initialized according to the participant’s age, sex, race, weight, and height by inputting or updating these measures in the Apple Health app. Apple Health and Fitness notifications (eg, stand reminders, goal completions, daily coaching, special challenges, and activity sharing notifications) will be disabled. Participants will download the TestFlight application—an Apple Developer app that allows for testing beta versions of our WWJ iOS and watchOS apps—and be sent an invitation email with a code to access the WWJ app. Participants will be guided through the WWJ app installation on the AW and paired iPhone. After downloading, the WWJ app goes through each data type, including precise location, which will be monitored to obtain user approval. Location services for the WWJ app will be turned to “always on and precise.” To familiarize participants with the WWJ app, visually enhanced instructions on how to tag precise geographic locations on the AW, use the Apple Calendar to schedule busy times on the iPhone, and restart the WWJ app will be provided in paper and digital forms, and tasks will be demonstrated on a laboratory iPhone and AW.

#### WWJ Observation Period, Just-in-Time, and JITAI Periods

For the next six weeks, participants will be instructed to (1) wear the AW on their nondominant wrist during all waking hours, (2) charge the AW during nonwaking hours, (3) tag all locations visited, (4) input and update busy times daily, (5) restart the WWJ app every morning upon waking, and (6) respond to all WWJ app feasibility notifications. During the 2-week observation period, the WWJ app will only monitor walking and stationary behaviors without sending any feasibility or intervention notifications. During the 2-week just-in-time learning period, the WWJ app will (1) monitor walking and SBs, (2) send intervention and feasibility notifications across all appropriate contexts, and (3) assess participant receptiveness to the intervention notifications to begin training for each participant’s unique contexts in which intervention notifications led to successful (vs failed) behavioral changes. During the 2-week JITAI period, the WWJ app will (1) monitor walking and SBs and contextual variables; (2) send notifications at the most opportune moments based on the decision tree policy and the extent to which prior notifications were successful [39]; (3) assess participant receptiveness; and (4) elicit reasons for nonreceptiveness.

#### Postintervention Visit

The postintervention visit will take place in a private room to complete postassessments (ie, behavior change constructs, anthropometrics, and resting vital signs), uninstall the WWJ app, and assist the participant in restoring all original settings on their iPhone and AW. If an AW was borrowed for this study, the device will be returned at this time.

### Analytic Plan, Sample Size, and Power Estimates

#### Data Processing

The WWJ server will store all data for manual processing. Data processing will start by ensuring all timestamps for walking and stationary bouts are sequentially aligned. This alignment will allow for accurate calculations for transitions between these states and other activity modes. Outliers, including device-specific artifacts, such as overlapping timestamps and erroneous bout durations such as those too short to be meaningful (eg, <10 seconds) or exceeding physical limits (eg, >5 hours of continuous walking) and walking speeds surpassing human capacities (eg, >3 m/s) [40], will be identified and reviewed for manual correction or exclusion. Additionally, geographic locations tagged in the data and any nonadherence responses marked as “other” and inputted by participants will be downloaded and coded using an inductive approach to group similar entries into cohesive categories emerging organically from the data. Once fully processed, all data will be stored in a standardized format.

#### Analytic Plan

Using the latest version of JMP Statistical Software (currently JMP, version 19.01, SAS Institute Inc, 1989-2025), preliminary analyses will include baseline descriptive statistics and exploratory data analysis. Baseline characteristics of the participants (eg, self-reported MVPA minutes from the screener, demographics, behavior change constructs, anthropometrics, and resting vital signs); recruitment, enrollment, and retention rates; implementation fidelity; adherence and compliance rates; user-burden; acceptability; treatment-specific expectations of benefits and preferences will be summarized in frequency tables for categorical variables and means and SDs for quantitative variables. Qualitative responses to applicable feasibility notifications and open-ended exit surveys will be explored.

Walking behavior and stationary time measures through all 3 periods—baseline, just-in-time, and JITAI—support a 1-way within-subjects ANOVA analysis of continuous measures (eg, average bout duration, speed, distance, and stationary time per study period for each participant) to demonstrate the feasibility of effecting a change in the behavior profile throughout the WWJ study. The intended 1-way within-subjects ANOVA assumes limited outliers, normality, and sphericity, which will be assessed graphically using boxplots, the Shapiro-Wilk test, and Mauchly test, respectively. Outliers, if present, will be assessed for their potential to weaken the analysis and removed if necessary. Nonnormal data will be transformed using the Box-Cox procedure to achieve normality. If sphericity is violated, the Greenhouse-Geisser and Huynh-Feldt corrections will be used. Tukey post hoc testing will be used to explain the differences between WWJ periods. If normality cannot be approximately achieved, the nonparametric Friedman test will be used. The model will be extended to allow for covariates if data screening of other collected demographic measures (eg, education) suggests their importance.

During implementation, communication drops or a lack of participant adherence may result in missing observations in some instances that inform the planned ANOVA. It will therefore be necessary to explore missing observations as to missing completely at random, missing at random, and missing not at random by using missing data analytics in JMP to associate instances with other study conditions (eg, software considerations and behavioral context). To the extent that the structure leading to the missing observation is not present or identifiable, missing at random or missing completely at random will be assumed for those records, and a linear mixed model will be used, leveraging maximum likelihood for estimation to achieve the within-subjects ANOVA goals without case-wise deletion. Missingness will be summarized in the analysis.

Secondary analyses will examine the initial effectiveness of the walking and stationary prompts. Specifically, the success or failure of notifications to encourage walking (ie, walking faster or longer) or reduce stationary time (ie, registered walking for at least 1 minute). Interarrival times and bouts of walking and stationary times computed at baseline will be summarized in terms of location and spread for continuous values or as frequency tables for bouts of activity.

### Sample Size and Power Estimates

Power calculations for the planned 1-way ANOVA were performed using G*Power Version 3.1.9.6. [34]. With 3 collapsed times of repeated measures (baseline, just-in-time, and JITAI) and assuming a within-subject correlation of 0.5, given the focus on feasibility for this study, the most effective sample size for predicting our primary outcomes (change in walking behavior and stationary time) was 17 participants with an α level of 0.05, medium-to-large effect size of Cohen f=0.32, and power of 0.8. Allowing for 15% attrition due to dropout or adherence issues, our proposed sample size is 20 participants.

### Ethical Considerations

This study protocol aligns with the ethical standards of the American Psychological Association and was approved by the University of Delaware Institutional Review Board (IRBNet ID: 1771712-9). All eligible participants will sign an informed consent form to participate. To protect user privacy and data security, all learning models are run locally on the AW via the WWJ app. Study participants will be compensated US $50.00 via an e-gift card voucher.

## Results

Participant recruitment and enrollment began in fall 2022. Data collection was completed in 2024 with a final study sample of 18 participants. Upon completion of the analyses, the results of this study are expected to be submitted for publication. This study was initially funded in March of 2020.

## Discussion

### Principal Findings

This protocol paper outlines the development of the WWJ server and app architecture and methodology for a pilot 6-week study to promote walking behavior and reduce stationary behavior among adults who are currently not meeting the 2020 WHO MVPA recommendations [5]. The objective of this study will be to examine the feasibility and initial impact of a 2-week JITAI approach on walking and stationary behaviors compared to a 2-week baseline and 2-week just-in-time approach. The cornerstone of a JITAI is the identification of decision points, decision rules, and contextual variables, which are taken into consideration for learning and adapting to provide potential behavioral interventions [24]. Our JITAI approach will identify and integrate both contextual variables and user receptiveness learned during the just-in-time period to provide user-specific intervention notifications to walk fast, walk longer, and stand up and move around at more personalized opportune times.

To provide support for behavior change, a JITAI will distinguish opportune times by predicting whether the person requires support, the type or amount of support needed, and whether or not the support will be acted upon [24,41]. Thus, user-specific receptiveness is a key feature of a JITAI [24]. For instance, a 2023 walking-based JITAI protocol accounted for receptiveness based on users’ previous walking behavior occurring within the same 3-hour time window [42]. Our JITAI extends upon this approach by including a 2-week observation and a 2-week just-in-time “learning” period to train the learner based on user receptiveness within contexts. This unique application of the just-in-time approach—sending intervention notifications whenever a participant meets predetermined thresholds, regardless of the likelihood of receptivity—serves to prune out nonopportune or “nonactionable” moments over time. In doing so, it enables greater customization based on user- and context-specific patterns of success and nonsuccess in promoting behavior change. For example, a 2016 walking-based just-in-time study found that delivering intervention notifications when participants “overused” their smartphone, were sedentary “for a long time,” were walking, or had “just had their meals” effectively increased walking behavior. Yet, participant feedback suggested that the system should be synced with their calendars to avoid sending intervention notifications at nonopportune—and potentially embarrassing—times [43]. Notably, this study also limited its sample to participants in the TTM’s contemplation and preparation stages [43]. In contrast, our dynamic approach will allow for continued learning opportunities during the JITAI by occasionally sending intervention notifications during previously “nonactionable” times. This policy decision assumes that a user’s state of need and receptivity may change over time, potentially enabling participants to progress fluidly through applicable stages of the TTM [44].

Similarly, our JITAI approach will not impose a minimum or maximum threshold for the frequency of intervention notifications. For instance, previous JITAIs have capped their maximum daily intervention notifications to delivering only 3 [45,46] or 4 [42] instances, regardless of whether additional opportune moments will arise. Additionally, in the 2023 walking-based JITAI, intervention notifications ceased once participants achieved a predetermined step-count goal [42]. Building on this evidence, our JITAI study will leverage a flexible approach by continuing to deliver intervention notifications even after participants meet the minimum recommendation of 150 weekly minutes of MVPA. This aligns with the WHO recommendation to encourage individuals to exceed the minimum activity thresholds for additional health benefits [5].

Furthermore, our approach extends upon the current literature by passively monitoring minute-level changes in activity patterns and their environmental contexts of busy schedules, location, and weather to personalize support. This contrasts with some previously published JITAIs that rely on self-reported states or ecological momentary assessments for policy decisions on whether to send an intervention notification [27]. For instance, a 2022 JITAI protocol aimed to reduce SB by sending intervention notifications based on preferred weather conditions, which were preidentified by participants during onboarding [47]. The system also considered nearby locations deemed suitable for PA by the researchers to send intervention notifications suggesting that the participant engage in PA rather than SB at one of the reachable locations [47]. In addition, a 2022 JITAI study aimed to promote PA by sending intervention notifications based on individual states aligned with predetermined participant-indicated preferred times of day and weather forecasts [48]. Our JITAI approach extends upon these protocols by considering passively-monitored location, time of day, real-time weather conditions—including precipitation, temperature, humidity, and wind speed—as well as external scheduling logistics (eg, “busy times” due to work restrictions or doctors’ appointments). These environmental contexts have been well-documented to impact PA and SB [49,50] and were deemed important to consider by the participants in the 2016 walking-based just-in-time study [43].

Nonetheless, the collection and use of such granular personal and environmental data may raise concerns surrounding data security and privacy [51]. To mitigate these concerns, it is essential to implement robust data protection measures for JITAIs, especially those that leverage users’ personal devices. To optimize data security and privacy, our WWJ app will run its learning models locally on the AW before securely sending and storing data on our server. Additionally, all transmitted data will be encrypted end-to-end, and participants’ data will only be matched to their participant ID to foster user trust and ultimately support greater engagement and adherence to the JITAI intervention.

### Strengths and Limitations

A key strength of our JITAI approach lies in using machine learning techniques to exclusively learn behavioral patterns, environmental contexts, and receptiveness with intervention notifications sent during the just-in-time learning period. This dynamic learning process has great promise to enhance the adaptability of the intervention and potential for behavior change by evolving to meet the unique support needs and preferences of each user. To date, this study is one of only a few true JITAI approaches in which a just-in-time learning period is leveraged to systematically assess, learn from, and dynamically adapt to user receptiveness and contextualized activity patterns in real time [24,27]. A second study strength is the assessment of theoretical psychosocial variables known to be associated with PA and SB behavior change—stages and processes of PA change, PA self-regulation, and PA self-efficacy. Exploring these variables may help identify relevant behavior change constructs to inform the development of a suitable framework for refining behavioral JITAI protocols. This framework could facilitate the proactive integration of psychosocial variables with real-time adaptive learning, further enhancing JITAI personalization.

Despite these strengths, we acknowledge the potential for limitations. One possible limitation is the extent to which participants’ engagement with this study influences the potential for the server to learn. For learning to occur, the system must detect the most opportune times to deliver behavioral prompts, which is dependent upon participants meeting the preset minimum activity thresholds. In this study, we set 250 meters as the minimum threshold for detecting walking bouts with viable opportunities to walk faster or longer. As such, participants who do not meet this minimum threshold may not receive any walking intervention notifications during the just-in-time or JITAI periods, and therefore, the potential for the server to learn is likely negligible for participants who do not engage in walking bouts below this threshold. In contrast, participants regularly meeting and exceeding the minimum thresholds will likely have more successful JITAI intervention notifications due to the potential for greater system learning. Although a unique feature of this study is the just-in-time learning period, based on previous literature, the extent to which the perceived user burden of a just-in-time “learning” approach may contribute to potential participant burnout or disengagement over time is unclear. Understanding these responses to intervention notifications is essential for implementing effective behavioral JITAIs that maximize learning, user engagement, and long-term adherence without overwhelming the user. While this study’s sample size of 20 participants is relatively small, the primary focus of this study will be establishing feasibility and acceptability, rather than generating generalizable behavioral outcomes. We expect the results and corresponding effect sizes will inform an adequately powered sample size for a future randomized controlled trial.

### Anticipated Findings and Future Directions

On Wednesday, March 13, 2024, the methodology for this work was presented as a poster at the Society of Behavioral Medicine’s 45th Annual Meeting and Scientific Sessions in Philadelphia, Pennsylvania, earning the Meritorious Award for an outstanding student-authored abstract submission and research presentation. This abstract was published in the *Annals of Behavioral Medicine* [52].

It is expected that our WWJ study will demonstrate adequate feasibility and acceptability. Following this preliminary examination, we expect to expand upon this approach by considering psychosocial constructs to ground our approach in a theoretical framework and additional contextual factors, such as personal affect, personality traits, or social factors, to further impact participant engagement and receptivity for this JITAI approach for adults who are inactive. This refinement could contribute significantly to the broader field of PA interventions, offering more tailored and impactful strategies for promoting long-term behavior change across diverse populations.

### Conclusions

In conclusion, the WWJ study represents an innovative step in personalized health interventions, combining real-time contextual data with adaptive notifications to promote walking behavior and reduce stationary time among adults who are currently inactive. By demonstrating feasibility and acceptability, this work lays the foundation for future research to leverage the just-in-time “learning” period to train JITAI approaches, allowing them to dynamically adapt to user receptiveness and contextualized activity patterns in real time. This approach holds promise for developing more personalized and scalable interventions, ultimately contributing to the broader goal of supporting long-term PA and SB behavior change.

## Abbreviations

API: application programming interface
AW: Apple Watch
BP: blood pressure
EE: energy expenditure
HR: heart rate
JITAI: Just-in-Time Adaptive Intervention
LPA: light-intensity physical activity
MET: metabolic equivalent of task
MPA: moderate-intensity physical activity
MVPA: moderate-to-vigorous-intensity physical activity
PA: physical activity
REDCap: Research Electronic Data Capture
SB: sedentary behavior
TTM: transtheoretical model
WHO: World Health Organization
WWJ: Walking With Just-in-Time Adaptive Interventions
